# Effect of tuberculosis infection on mortality of HIV-infected patients in Northern Tanzania

**DOI:** 10.1186/s41182-020-00212-z

**Published:** 2020-04-27

**Authors:** Edson W. Mollel, Jim Todd, Michael J. Mahande, Sia E. Msuya

**Affiliations:** 1 Institute of Public Health, Department of Epidemiology and Biostatistics, Kilimanjaro Christian Medical University College (KCMUCo), Moshi, Tanzania; 2 Northern Zone Blood Transfusion Centre, P.O.BOX 823 Kilimanjaro, Tanzania; 3 Department of Population Health, London School of Hygiene and Tropical Medicine, London, UK; 4 Institute of Public Health, Department of Community Health, Kilimanjaro Christian Medical University College, Kilimanjaro, Tanzania; 5 Department of Community Health, Kilimanjaro Christian Medical Centre (KCMC), Kilimanjaro, Tanzania

**Keywords:** Tuberculosis, HIV, Tanzania, Mortality rates, sub-Saharan Africa

## Abstract

**Background:**

TB and HIV are public health problems, which have a synergistic effect to each other. Despite the decreasing burden of these two diseases they still make a significant contribution to mortality. Tanzania is among the 30 high TB and HIV burden countries.

**Methods:**

Routine data over 6 years from people living with HIV (PLHIV) attending health facilities in three regions of Northern Tanzania were analyzed, showing mortality trends from 2012 to 2017 for HIV and HIV/TB subpopulations. Poisson regression with frailty model adjusting for clustering at health facility level was used to analyze the data to determine mortality rate ratios (RR) and 95% confidence intervals (95%CI).

**Results:**

Among all PLHIV the overall mortality rate was 28.4 (95% CI 27.6–29.2) deaths per 1000 person-years. For PLHIV with no evidence of TB the mortality rates was 26.2 (95% CI 25.4–27.0) per 1000 person-years, and for those with HIV/TB co-infection 57.8 (95% CI 55.6–62.3) per 1000 person-years. After adjusting for age, sex, residence, WHO stage, and bodyweight, PLHIV with TB co-infection had 40% higher mortality than those without TB (RR 1.4; 95% CI 1.24–1.67).

**Conclusions:**

Over the 6-year period mortality rates for HIV/TB patients were consistently higher than for PLHIV who have no TB. More efforts should be directed into improving nutritional status among HIV patients, as it has destructive interaction with TB for mortality. This will improve patients’ body weight and CD4 counts which are protective against mortality. Among PLHIV attention should be given to those who are in WHO HIV stage 3 or 4 and having TB co-infection.

## Introduction

TB and HIV are public health problems, which have a synergistic effect to each other. In people living with HIV (PLHIV), TB increases HIV replication and viral heterogeneity [17, 18, 26, 36]. HIV on the other hand lowers the immunity against TB leading to increased active TB infection, re-infection, or reactivation. It also increases the risk of TB progression from latent TB to active TB disease [9, 10, 12, 27, 33]. The burden of TB in countries with a high HIV prevalence, like Tanzania, is substantial. Globally, over the last two decades, the incidence of TB has declined from an incidence of 172 cases per 100,000 populations in year 2012 to 132 cases per 100,000 in year 2018 probably due to increased coverage and efficient TB management, HIV awareness and widespread use of antiretroviral therapy (ART) [13, 42]. Early TB diagnosis, especially with the advent of molecular diagnostic tests at HIV clinics, has led to early TB treatment, which has contributed to reducing mortality rates for TB, even in high HIV settings [37, 40]. Education on TB, and good collaborative HIV and TB activities, with increased use of Isoniazid Preventive Therapy (IPT) among PLHIV, have also contributed to reduced mortality rates [6, 19, 28, 39].

HIV and TB make a significant contribution to mortality with 770,000 people dying of AIDS related causes in 2018 and 1.6 million people dying of TB in 2017, with 300,000 deaths among those with HIV/TB co-infection [38, 41]. Sub-Saharan Africa (SSA) accounts for 71% of the global HIV/TB burden, and Tanzania is among the 30 countries with the highest burden of TB and HIV [42]. HIV/TB co-infected patients have 1.8 times higher risk of mortality compared to those who are HIV-infected TB-free patients [4]. The odds of mortality are 3.5 times higher when the patients have HIV/Drug Resistant Tuberculosis co-infection [23]. In 2018, Tanzania reports showed that there were 22,000 deaths among HIV negative TB patients and 16,000 deaths among HIV positive patients co-infected with TB [42]. HIV/TB treatment success rate is only 85% in Tanzania, meaning the remaining 15% have poor treatment outcomes [41].

Factors that have been associated with increased mortality among HIV/TB co-infected patients include low CD4 count, WHO HIV stages 3 or 4, not receiving ART, and not being on cotrimoxazole prophylaxis therapy (CPT), being female sex worker, older age and being bed-ridden [1, 32, 35]. Factors that have been associated with mortality among HIV-positive TB-negative patients include WHO HIV stage 3 or 4, older age, low CD4 counts, low hemoglobin, low educational status, bodyweight, as well as low adherence to ART [5, 8, 29, 30]. These factors have shown to differ between settings.

The World Health Organization (WHO)’s End TB Strategy goal is to reduce the number of TB deaths by 35% in 2020 and by 90% by 2030 [42]. Although there has been a reduction in TB deaths by only 11% globally and 16% in the African region TB deaths Tanzania is on track to achieve this milestone with a 30% reduction [42]. Many studies have reported risk factors for mortality in PLHIV and PLHIV who are co-infected with TB. However it is not yet known how mortality rates for PLHIV, and PLHIV who are co-infected with TB, have changed over time, and whether the factors associated with mortality differ between those co-infected with TB and those who are not TB infected [42].

This study aimed to compare trends of mortality for HIV and HIV/TB subgroups over six-year period, from 2012 to 2017. Hence, describing the transition period from the era of Millennium Development Goals (MDGs) to Sustainable Development Goals (SDGs). It will inform policy makers on the magnitude of the problem and enable them to devise potential interventions to tackle it. The study has used routinely collected data from a big database maintained by the Ministry of Health Community Development Gender Elderly and Children (MoHCDGEC) which will enable different insights to other studies on HIV/TB mortality.

## Methods

### Study design and settings

This was a retrospective cohort using routinely collected data on PLHIV attending HIV services at health facilities from 1st January 2012 to 31st December 2017 from the three regions of Northern Tanzania, namely, Arusha, Tanga, and Kilimanjaro. HIV services are provided in Care and Treatment Centres (CTCs) which enrol PLHIV following HIV diagnosis, provide ART, and monitor patient progress. In Tanzania, PLHIV are expected to attend CTC every month for a check-up and to receive their ART and other medication. A standardized MoHCDGEC-authorized individual CTC patient record is completed on every occasion the PLHIV visits the CTC. In this analysis, data came from CTC at all levels (dispensary, health centres and hospitals) and from both private and public facilities in three regions of Tanzania. At every visit TB screening is undertaken and those positive undertake further tests to make a confirmed TB diagnosis [21, 22, 24]. This study used the individual patient records from all CTC that entered patient visit data into the national CTC national database.

### Study population

All PLHIV attending CTCs in these three regions who were 15 or more years of age, and attended for care between 1st January 2012 to 31st December 2017, were eligible for inclusion in this analysis.

### Study variables

The individual CTC patient record routinely collects data at enrollment on age, sex, marital status, weight, nutritional status WHO HIV clinical stage and functional status. Functional status has three categories: those capable of working, those who cannot work but are ambulatory, and those who are bedridden. Nutritional status was subjectively evaluated by the health care worker, who categorized patients into three categories: normal, moderate, or severe under-nutrition. At each visit to the CTC, the patient record is used to record details on the ART regime, using defined MoHCDGEC codes and categorized into first-line and second-line ART regimes for the purpose of this analysis. CD4 counts were taken when needed, but were discontinued in 2016 when viral load measurements were recommended for monitoring patient progress.

### Data analysis

After data were de-identified and cleaned, they were analyzed using statistical software package, STATA 15. Categorical data were summarized as frequencies and percentages. Continuous variables were summarized using median and interquartile range (IQR) or using mean and standard deviation. The key dependent variable was mortality from any cause. The start time for the person-time at risk was taken to be from 1 January 2012 or date of first enrollment at CTC (if that was later that 1 January 2012). End study time was defined as the date of death of any cause, or for those who did not die, the date last seen at CTC, or 31 December 2017 if they attended CTC after that date. TB diagnosis was defined as having a record for starting anti-TB medications. Patients who were positive to the TB screening questions, but did not have a confirmed TB diagnosis were defined as non-TB cases. A confirmed diagnosis could be made through chest radiography, microbiology culture, PCR (eg GeneXpert) or clinical judgement [25]. In Tanzania, PLHIV are treated with triple therapy composed of either 2 of any Nucleoside Reverse Transcriptase Inhibitors (NRTIs) and 1 Non-Nucleoside Reverse Transcriptase Inhibitors (NNRTIs), or 2 NRTIs and 1 Protease Inhibitors (PIs).

**Fig. 1:**
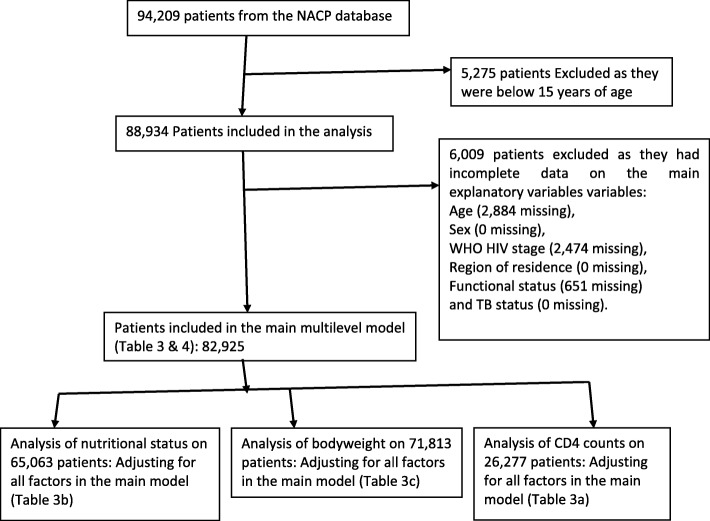
The study’s flow diagram

Mortality rates among HIV patients and HIV/TB patients over a period of 6 years were determined for different socio-demographic and clinical characteristics. Variables with few missing data were analyzed as complete case records, while for CD4 count and nutritional status, which had more than 10% missing data, a restricted analysis was performed for each variable separately (Fig. 1). The trends of mortality from 2012 to 2017 were determined and compared between HIV and HIV/TB subpopulations. Cluster level analysis using (analysis of variance) ANOVA was done to determine the effects of health facilities on HIV mortality. Poisson regression models were used to determine rate ratios (RR) and 95% confidence intervals (95%CI), with a multilevel fraility component to adjust for clustering by health facility. Finally, Poisson regression models with frailty were used to determine the interactions between TB co-infection and other factors on mortality among PLHIV.

## Results

The study involved 88,934 patients who were HIV positive, of which 83,489 (93.9%) had no TB co-infection and 5,446 (6.1%) had HIV/TB co-infection at some stage over the 6 years. There were 25,618 (28.8%) male patients and 29,007 (32.6%) were aged between 35 and 44 years of age when they were started to be followed up. Among those who had their CD4 count measured, 17,835 (63.7%) of them had CD4 count below 350 cells/ul. Most patients were from Tanga, 45,095(50.7%) than from Arusha or Kilimanjaro regions (Table 1).

**Table 1:** Mortality rates by sociodemographic and clinical characteristics at enrolment into HIV services for PLHIV patients in three regions of Northern Tanzania (*N* = 88,934)

	Number of PLHIV (%)	Number of deaths for all PLHIV (%)	Person-years of follow-up (in 1000 years)	Mortality rate per 1000 for PLHIV (95% CI)
Characteristics	88,934	4,757 (5.3)	167.7	28.4 (27.6–29.2)
Age (*N* = 86,050)				
• 15–24 • 25–34 • 35–44 • 45–55 • Above 55	8070 (9.4) 23,648 (27.5) 29,007 (33.7) 16,906 (19.6) 8419 (9.8)	258 (3.2) 853 (3.6) 1600 (5.5) 1147 (6.8) 899 (10.7)	10.25 31.99 59.71 42.46 23.29	25.2 (22.3–28.4) 26.7 (24.9–28.5) 26.8 (25.5–28.1) 27.0 (25.5–28.6) 38.6 (36.2–41.2)
Sex (*N* = 88,934)				
• Male • Female	25,618 (29.1) 63,316 (70.9)	1906 (7.4) 2851 (4.5)	45.23 122.46	42.1 (40.3–44.1) 23.3 (22.4–24.2)
Marital status (*N* = 82,241)				
• Cohabiting • Divorced • Married • Single • Widow/widower	1161 (1.4) 8067 (9.8) 43,603 (53.0) 23,298 (28.3) 6112 (7.4)	51 (4.4) 530 (6.6) 2204 (5.1) 1345 (5.8) 410 (6.7)	2.23 16.99 85.50 41.99 13.36	22.9 (17.4–30.1) 31.2 (28.7–34.0) 25.8 (24.7–26.9) 32.0 (30.4–33.8) 30.7 (27.9–33.8)
Region (*N* = 88,934)				
• Arusha • Kilimanjaro • Tanga	14,316 (16.1) 29,524 (33.2) 45,094 (50.7)	517 (3.6) 1293 (4.4) 2947 (6.5)	16.21 54.02 97.47	31.9 (29.3–34.8) 23.9 (22.7–25.3) 30.2 (29.2–31.3)
TB status (*N* = 88,934)				
• No TB • TB co-infection	83,488 (93.9) 5446 (6.1)	4086 (4.9) 671 (1.2)	156.08 11.61	26.2 (25.4–27.0) 57.8 (53.6–62.3)
Body weight (*N* = 83,378)				
• Below 40 kg • 40–60 kg • Above 60 kg	7,993 (9.6) 49,815 (59.7) 25,570 (30.7)	691 (8.6) 3014 (6.1) 790 (3.1)	10.90 97.19 51.15	63.4 (58.9–68.3) 31.0 (29.9–32.1) 15.4 (14.4–16.6)
HIV WHO stage (*N* = 86,460)				
• Stage 1 • Stage 2 • Stage 3 • Stage 4	22,238 (25.7) 19,914 (23.0) 32,348 (37.4) 11,960 (13.8)	482 (2.2) 940 (4.7) 2091 (6.5) 1159 (9.7)	29.82 38.35 70.83 25.55	16.2 (14.8–17.7) 24.5 (23.0–26.1) 29.5 (28.3–30.8) 45.4 (42.8–48.0)
CD4 categories (*N* = 28,013)				
• Below 350 • 350-500 • Above 500	17,835 (63.7) 4756 (17.0) 5422 (19.4)	1479 (8.3) 157 (3.3) 100 (1.8)	34.19 8.71 7.87	43.3 (41.1–45.5) 18.0 (15.4–21.1) 12.7 (10.4–15.5)
Functional status (*N* = 88,283)				
• Bedridden • Ambulatory • Working	4199 (4.8) 590 (0.7) 83,494 (94.6)	503 (12.0) 78 (13.2) 4152 (5.0)	7.14 0.58 159.02	79.5 (64.6–76.9) 135.6 (108.6–169.3) 26.1 (25.3–26.9)
Nutritional status (*N* = 68,864)				
• Ok • Moderate • Severe	63,626 (92.4) 4843 (7.0) 395 (0.6)	3237 (5.1) 511 (10.6) 53 (13.4)	113.89 8.44 0.42	28.4 (27.5–29.4) 60.6 (55.5–66.1) 125.6 (95.9–164.3)
ARV adherence (*N* = 46,438)				
• Good adherence • Poor adherence	45,281 (97.5) 1157 (2.5)	2403 (5.3) 127 (11.0)	109.7 2.7	21.9 (21.0–22.8) 46.6 (39.2–55.5)
ARV regimen (*N* = 51,525)				
• First line • Second line	50,314 (97.6) 1211 (2.4)	2669 (5.3) 73 (6.0)	111.0 3.21	24.0 (23.2–25.0) 22.7 (18.1–28.6)

A total of 4757 HIV-positive patients died during 167,700 person-years of follow-up, giving the overall mortality rate of 28.4 (95% CI 27.6–29.2) per 1000 person-years. The mortality rate was 26.2 (95% CI 25.4–27.0) per 1000 person-years among PLHIV who had no TB, and 57.8 (95% CI 53.6–62.3) per 1000 person-years among those with HIV/TB co-infection (Table 1). The highest mortality rates were among patients who were ambulatory (Mortality rate of 135.6 (95% CI 108.6–169.3) per 1000 person-years), had severe under-nutrition (125.6 (95% CI 95.9–164.3) per 1000 person-years), bedridden (79.5 (95% CI 64.6–76.9) per 1000 person-years) and with moderate under-nutritional (60.6 (95% CI 55.5–66.1) per 1000 person-years).

**Fig. 2:**
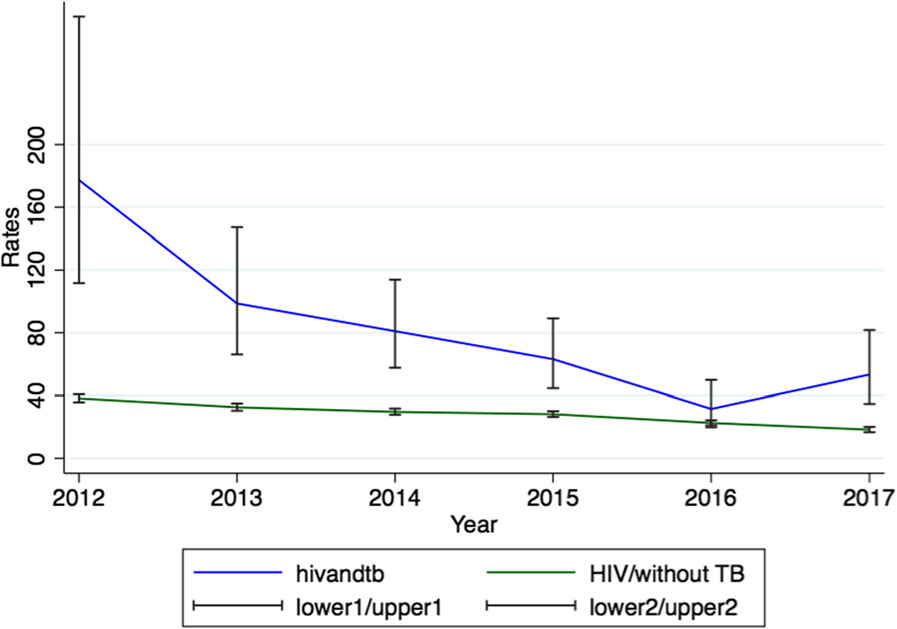
Trends of mortality over the years per 1000 person-years for HIV and HIV/TB co-infected patients

HIV/TB co-infected patients had a mortality rate of 177.3 (95% CI 111.7–281.5) per 1000 person-years in 2012 which declined to 31.5 (95% C1 19.8–49.9) per 1000 person-years in 2016 and 53.3 (95% CI 34.7–81.7) per 1000 person-years in 2017. For PLHIV who had no TB history mortality rates also declined from 38.2 (95% CI 35.7–40.8) per 1000 person-years in 2012 to 18.3 (95%CI 16.7–20.1) per 1000 person-years in 2017 (Fig. 2).

Using ANOVA analysis on the mortality rates by health facilities showed differences across health facility types. PLHIV attending health centers and hospitals had higher mortality rates than those attending a dispensary (Table 2). Mortality among PLHIV attending private facilities was higher than those attending a public health facility. There was also differences between the regions with the highest mortality in Arusha region (41 deaths per 1000 person-years) and the lowest in Tanga region (33 patients deaths per 1000 person-years) (Table 2).

**Table 2:** Comparison of cluster level’s mortality rates per 1000 person-years among PLHIV in 253 health facilities in three regions of Northern Tanzania (*N* = 88,934)

				Mortality rates per 1000 person-years	
Cluster variables	Number of deaths	Number of clusters with at least 1 death	Average number of deaths per cluster	Average (SD) mortality rate	*p* value (ANOVA) for mortality rates over cluster levels
Facility types					
• Dispensary • Health centers • Hospitals • Overall	338 2200 2219 4757	47/72 114/117 65/66 226/253	6.6 37.4 90.0 42.7	20 39 39 33	< 0.000
Facility ownership					
• Private • Public • Overall	744 4013 4757	57/58 169/195 226/253	36.7 44.4 42.7	47 30 33	< 0.000
Region					
• Arusha • Kilimanjaro • Tanga • Overall	517 1293 2947 4757	61/74 64/73 101/106 226/253	18.3 36.4 65.0 42.7	41 27 33 34	0.005

A Poisson regression model controlling for age, sex, bodyweight, WHO HIV stage, region, functional status, and TB status with a multilevel component analysis using health facility as the cluster variable was used (Table 3). Compared to those aged 15–24 years higher mortality was observed among PLHIV aged between 35 and 44 years (RR = 1.30, 95%CI 1.05–1.61) and those aged above 55 years (RR = 1.88, 95%CI 1.50–2.37). Those with moderately (RR = 1.61, 95%CI 1.39–1.85) and severely (RR = 1.90, 95% CI 1.24–2.90) poor nutritional status had higher mortality compared to those with normal nutritional status. Conversely, lower mortality was observed among females (RR = 0.62, 95% CI 0.56–0.69) compared to males, body weight between 40 and 60 kg (RR = 0.50, 95%CI 0.43–0.58) and above 60 kg (RR = 0.26, 95%CI 0.22–0.31) compared to those under 40 kg in weight. A lower mortality was observed in Tanga region (RR = 0.78, 95% CI 0.69–0.91) compared to Arusha. PLHIV with TB co-infection had higher mortality (RR = 1.4, 95%CI 1.24–1.67) compared to those who had not experienced TB infection.

**Table 3:** Poisson regression with multilevel analysis (health facility as cluster variable) of the determinants of mortality rates among PLHIV in Northern Tanzania (*N* = 82,925)

Characteristics	Survival status Event (death) censored		Crude rate ratio (95%CI)	Adjusted rate ratio (95%CI) adjusting only for clusters	ARR (95% CI) adjusting for other factors and for clusters *
Age					
15–24	258	7812	1	1	1
25–34	853	22,795	1.03 (0.90–1.19)	1.06 (0.92–1.22)	1.21 (0.97–1.51)
35–44	1600	27,407	1.14 (1.00–1.31)	1.17 (1.03–1.34)	1.30 (1.05–1.61)
55–55	1147	15,759	1.21 (1.05–1.39)	1.24 (1.08–1.43)	1.23 (0.98–1.54)
Above 55	899	7520	1.90 (1.64–2.19)	1.92 (1.66–2.21)	1.88 (1.50–2.37)
Sex					
Male	1906	23,712	1	1	1
Female	2851	60,465	0.57 (0.54–0.60)	0.57 (0.54–0.60)	0.62 (0.56–0.69)
HIV WHO stage					
Stage 1	482	21,756	1	1	1
Stage 2	940	18,974	1.45 (1.30–1.61)	1.51 (1.36–1.68)	1.38 (1.13–1.69)
Stage 3	2091	30,257	1.74 (1.59–1.92)	1.90 (1.73–2.09)	1.58 (1.32–1.90)
Stage 4	1159	10,801	2.70 (2.43–2.99)	3.01 (2.75–3.39)	2.08 (1.71–2.54)
Region					
Arusha	517	13,799	1	1	1
Kilimanjaro	1293	28,231	0.73 (0.66–0.80)	0.76 (0.69–0.85)	0.88 (0.74–1.03)
Tanga	2946	42,148	0.93 (0.85–1.02)	0.95 (0.87–1.04)	0.78 (0.69–0.91)
Functional status					
Bedridden	503	3696	1	1	1
Ambulatory	78	512	1.90 (1.51–2.39)	1.96 (1.56–2.47)	3.59 (2.39–5.39)
Working	4152	79,342	0.38 (0.34–0.41)	0.38 (0.35–0.42)	0.56 (0.47–0.68)
TB status					
No TB	4085	79,403	1	1	1
TB Co-infection	671	4775	2.23 (2.06–2.41)	2.25 (2.08–2.44)	1.4 (1.24–1.67)
Nutritional status **					
Ok	3237	60,389	1	1	1
Moderate	511	4332	2.11 (1.93–2.31)	2.06 (1.90–2.28)	1.61 (1.39–1.85)
Severe	53	342	4.06 (3.15–5.24)	4.15 (3.21–5.36)	1.90 (1.24–2.90)
Body weight***					
Below 40 kg	691	7302	1	1	1
40–60 kg	3014	46,801	0.77 (0.72–0.83)	0.77 (0.72–0.83)	0.50 (0.43–0.58)
Above 60 kg	790	24,780	0.39 (0.35–0.42)	0.39 (0.36–0.43)	0.26 (0.22–0.31)
CD4 count****					
Below 350	1479	16,356	1	1	1
350–500	157	4599	0.41 (0.35–0.48)	0.42 (0.36–0.49)	0.48 (0.39–0.58)
above 500	100	5322	0.31 (0.25–0.37)	0.31 (0.26–0.38)	0.40 (0.32–0.51)

*Adjusted for age, sex, region, WHO stage, functional status, TB status (*N* = 77,369)

**Adjusted for age, sex, region, WHO stage, functional status, TB status (*N* = 65,063)

***Adjusted for age, sex, region, WHO stage, functional status, TB status (*N* = 71,813)

****Adjusted for age, sex, bodyweight, region, WHO stage, functional status, TB status (*N* = 26,277)

There were interactions between TB and other independent variables with weaker evidence of differences in mortality among PLHIV who had TB compared to the rate ratios in PLHIV who did not experience TB (Table 4). The interaction was statistically significant for WHO HIV stage (*p* value = 1.49, 95% CI 1.01–2.19) and nutritional status (based on the subset of the data with nutritional status recorded). Among PLHIV co-infected with TB, those residing in Kilimanjaro region (RR = 0.63, 95%CI 0.46–0.86), and in Tanga Region (RR = 0.61, 95%CI 0.48–0.78) had lower mortality compared to Arusha region, and these effects were bigger than the effects seen in PLHIV who did not have TB.

**Table 4:** Poisson regression with multilevel analysis, with health facility as cluster variable in Northern Tanzania. Interaction effects for mortality among patients with HIV adjusted for the significant effects included in Table 3 (*N* = 82,925)

Characteristics	ARR (95%CI) PLHIV without TB	ARR (95%CI) PLHIV/TB co-infection *	Effect of TB infection in baseline **
Age			
15–24	1		2.06 (1.42–2.99)
25–34	1.27 (1.07–1.52)	0.97 (0.66–1.43)	
35–44	1.36 (1.14–1.61)	0.91 (0.63–1.33)	
45–55	1.34 (1.12–1.60)	0.93 (0.63–1.37)	
Above 55	1.98 (1.65–2.37)	1.26 (0.84–1.90)	
Sex			
Male	1		1.41 (1.22–1.62)
Female	0.57 (0.53–0.61)	0.63 (0.53–0.75)	
Body weight			
Below 40 kg	1		0.89 (0.70–1.13)
40–60 kg	0.46 (0.42–0.52)	0.80 (0.62–1.02)	
Above 60 kg	0.22 (0.20–0.26)	0.58 (0.42–0.80)	
HIV WHO stage			
Stage 1	1		1.49 (1.01–2.19)
Stage 2	1.35 (1.19–1.53)	1.53 (0.99–2.36)	
Stage 3	1.50 (1.33–1.68)	1.42 (0.96–2.09)	
Stage 4	2.22 (1.95–2.54)	1.74 (1.16–2.62)	
Region			
Arusha	1		2.02 (1.58–2.59)
Kilimanjaro	0.89 (0.77–1.03)	0.63 (0.46–0.86)	
Tanga	0.86 (0.76–0.97)	0.61 (0.48–0.78)	
Functional status			
Bedridden	1		1.15 (0.90–1.48)
Ambulatory	2.57 (1.88–3.52)	1.11 (0.54–2.31)	
Working	0.59 (0.52–0.67)	0.81 (0.64–1.03)	
Nutritional status			
Ok	1		1.61 (1.45–1.79)
Moderate	1.67 (1.50–1.86)	1.28 (1.03–1.61)	
Severe	2.40 (1.71–3.35)	0.63 (0.35–1.12)	
Year of follow-up			
2012	1		1.36 (1.05–1.74)
2013	0.83 (0.74–0.93)	0.87 (0.63–1.20)	
2014	0.73 (0.65–0.82)	0.91 (0.68–1.24)	
2015	0.71 (0.64–0.80)	0.71 (0.52–0.97)	
2016	0.56 (0.50–0.63)	0.46 (0.33–0.64)	
2017	0.44 (0.38–0.51)	0.72 (0.51–1.01)	

*ARR* adjusted rate ratio

Estimates of interaction effects are adjusted for age, sex, bodyweight, HIV WHO stage, region, functional status, and year of follow-up

*At non-baseline levels the effects are shown within each group (HIV alone and HIV/TB) comparing the level with the baseline level

**Adjusted rate ratios in the baseline level of each factor show the effect of TB on mortality at that baseline level

## Discussion

Mortality among PLHIV co-infected with TB is 1.4 times higher than in PLHIV who do not have TB. Since 2012 this increased mortality rate ratio has persisted for every year, although mortality rates for both groups have declined consistently since 2012. In PLHIV without TB mortality rates have declined by 52% over the 6 years from 38.2 deaths per 1000 person-years in 2012 to 18.2 deaths per 1000 person-years in 2017. In PLHIV co-infected with TB mortality has declined by 70% over the 6 years from 177.3 deaths per 1000 person-years in 2012 to 53.3 deaths per 1000 person-years in 2017. WHO reports have shown similar declines in mortality in other high TB burden countries, such as Central African Republic, Congo, Kenya, Liberia, Mozambique, Namibia, and Thailand [42]. The provision of ART for PLHIV has been very effective in reducing mortality, and PLHIV co-infected with TB have benefited more from these interventions as they had a higher mortality to start with.

The mortality in PLHIV co-infected with TB is greater than mortality in PLHIV without TB in every subgroup analyzed, with a Crude Rate Ratio for TB co-infected patients of 2.23. The mortality rates showed evidence of clustering by health facility, and a fraility model was fitted to adjusting for that clustering. The higher mortality rate ratio for PLHIV co-infected with TB persisted to be 2.2 times higher compared to those with TB co-infection after adjusting for health facility clustering. However, the effect was confounded by functional status and region, and after controlling for these and other factors the rate ratio decreased to around 1.4 times higher compared to those with no TB co-infection. This change may be partly due to the effect TB has on the functional status of PLHIV.

The significant reduction in mortality among PLHIV observed from 2012 to 2016 is probably due to increasing use of ART [22, 24]. UNAIDS reported in 2017 that in Northern Tanzania there had been only 10% reduction in TB mortality (from 63.1 death per 1000 person-years in 2015 to 53.3 deaths per person-years), but over the whole of Tanzania the reduction was between 25 and 49% [38]. The United Nation high level meeting conducted in 2016 set the target of reducing TB deaths among HIV patients by 35% in 2020 using 2015 data as baseline data [38]. As many TB deaths are contributed by PLHIV co-infected with TB, a delay or slow reduction in mortality in this group could negatively affect the achievement of other targets set by the WHO through the WHO End TB Strategy. The WHO End TB Strategy aims to reduce the absolute number of deaths due to TB by 35% by the end of 2020 [42]. Recent WHO data have also shown that Tanzania as a whole is on track to achieve this milestone as well [42].

In addition, isoniazid prevention therapy (IPT) to prevent TB infection was introduced in Tanzania in the late 2011 [28]. Increasing ART coverage among PLHIV has the potential to decrease TB progression among those co-infected with TB [2]. The introduction of a molecular diagnostic test, GeneXpert MTB/RIF, in 2013, which has high accuracy, has helped early TB detection and has facilitated early treatment initiation [22–24, 34, 40]. Increased awareness of TB and improved health systems to deal with TB infection are other important factors that have contributed to this success [6, 19].

Among study population, mortality rates were higher for patients with severely poor nutritional status, bed-ridden patients, and ambulatory patients. Similar findings have been observed in Dar es Salaam and Manyara in Tanzania, and in Cameroon and Ethiopia [7, 16, 20, 31]). Poor nutrition on the other hand is a risk factor for TB incidence among HIV patients, leading to a fatal vicious cycle [22, 24].

The study has also found that more deaths among HIV-positive patients are observed in hospitals and health centers compared to dispensaries. This can be attributed to the referral mechanism in the country, whereby patients with poor prognosis at the dispensary (which is a lower level facility) will always be referred to health center or hospital for further advanced management. Also, most seriously sick patients go to the hospitals and this is expected to increase the number of deaths in the hospitals. More deaths were observed in private hospitals than public hospitals, probably because most private facilities are hospitals rather than dispensaries. Regional differences in mortality rates can be attributed to several geographical inequalities (such as access to health facilities, availability of adequate health care providers, health seeking behaviors, etc.) despite the availability of the national guidelines and roll out of HIV and TB service. The contextual difference can be appreciated by the cluster effects which show that more than 6% of the variability in mortality is due to differences between health facilities across the different regions.

Several factors that have been associated with mortality among HIV patients have been observed elsewhere. Age above 55 years, HIV stage 3 and 4, and poor nutritional status have been associated with HIV mortality in Ethiopia [30] and China [14]. Other studies have found factors such as anemia and thrombocytopenia, delayed diagnosis, pneumonia as well as low adherence to ARVs have contributed to higher mortality [11, 14, 16, 30]. In our study, CD4 count higher than 350 cell/ul, body weight higher than 40 kg, and being female were protective against HIV mortality, similar findings have been observed elsewhere [3, 15].

For PLHIV, there were some interactions between TB and all other independent factors for mortality. This means that TB modifies the influence of these different factors on mortality. However, all factors had interactions with TB, but TB reduced the effect of functional status and poor nutrition on mortality, probably as there is a close correlation between these factors and TB infection.

This study’s strength is it used programmatically and routinely collected data from all health facilities in three regions for six years. It was limited in that TB diagnosis was defined as being on anti-TB treatment. With the potential of having inadequate TB diagnostic tools especially in lower facilities as well as delay of starting TB treatment since its detection, its very possible that the number of HIV/TB cases have been underestimated.

## Conclusions

Despite the study limitations, it shows that more efforts are needed to reduce the mortality rates among HIV patients, especially those with TB co-infection. Efforts should be directed into improving nutritional status and functional status among HIV patients, and avoiding advanced HIV disease through early identification of those infected and early use of ART. These factors are associated with higher mortality and are also risk factors for TB incidence. Improved nutritional status will also improve body weight and CD4 counts which are protective against mortality.

## Data Availability

Study’s data can be accessed from EWM after permission and approval from the NACP and the Government of Tanzania

## Abbreviations

ANOVA: Analysis of variance
ARR: Adjusted rate ratio
ART: Anti-retroviral therapy
ARV: Anti-retroviral
CD4: Cluster of differentiation 4
CI: Confidence interval
CPT: Cotrimoxazole prophylaxis therapy
CREC: College Research and Ethical Committee
CTC: Care and Treatment Centres
HIV: Human immunodeficiency virus
IPT: Isoniazid preventive therapy
IQR: Interquartile range
MDG: Millennium Development Goal
NACP: National AIDS Control Program
NNRTI: Non-Nucleoside Reverse Transcriptase Inhibitors
NRTI: Nucleoside Reverse Transcriptase Inhibitors
PLHIV: People living with HIV
PI: Protease inhibitors
SDG: Sustainable Development Goal
TB: Tuberculosis
UNAIDS: The Joint United Nations Programme on HIV and AIDS
WHO: World Health Organization

## References

[1] Abrha H, et al. Survival experience and its predictors among TB/HIV co-infected patients in Southwest Ethiopia. Epidemiology. 2015;5(3).

[2] Alene KA, Nega A, Taye BW (2013). Incidence and predictors of tuberculosis among adult people living with human immunodeficiency virus at the University of Gondar Referral Hospital, Northwest Ethiopia. BMJ Open.

[3] Alibhai A, Kipp W, Saunders LD, Senthilselvan A, Kaler A, Houston S (2010). Gender-related mortality for HIV-infected patients on highly active antiretroviral therapy (HAART) in rural Uganda. Int J Womens Health.

[4] Bassett IV, Chetty S, Wang B (2012). Loss to follow-up and mortality among HIV-infected people co-infected with TB at ART initiation in Durban, South Africa. J Acquir Immune Defic Syndr..

[5] Bereket D, Bezatu M, Tadesse A (2015). Survival and determinants of mortality in adult HIV/Aids patients initiating antiretroviral therapy in Somali Region, Eastern Ethiopia. Pan Afr Med J.

[6] Bisallah CI, Rampal L, Lye M-S, Mohd Sidik S, Ibrahim N, Iliyasu Z (2018). Effectiveness of health education intervention in improving knowledge, attitude, and practices regarding Tuberculosis among HIV patients in General Hospital Minna, Nigeria - A randomized control trial. PloS One.

[7] Biset AM (2017). Mortality and its predictors among HIV infected patients taking antiretroviral treatment in Ethiopia: a systematic review. AIDS Res Treat.

[8] Brien DO (2016). Risk factors for mortality during antiretroviral therapy in older populations in resource-limited settings. J Int AIDS Soc.

[9] Charalambous S, Grant AD, Moloi V (2008). Contribution of reinfection to recurrent tuberculosis in South African gold miners. Int J Tuberc Lung Dis.

[10] Daley CL, Small PM, Schecter GF (1992). An outbreak of tuberculosis with accelerated progression among persons infected with the human immunodeficiency virus. An analysis using restriction-fragment-length polymorphisms. N Engl J Med.

[11] Gunda DW, Nkandala I, Kilonzo SB, Kilangi BB, Mpondo BC (2017). Prevalence and risk factors of mortality among adult HIV patients initiating ART in rural setting of HIV care and treatment services in north western Tanzania: A retrospective cohort study. J Sex Transm Dis..

[12] Holmes CB, Wood R, Badri M (2006). CD4 decline and incidence of opportunistic infections in Cape Town, South Africa: implications for prophylaxis and treatment. J Acquir Immune Defic Syndr.

[13] Holtgrave DR, Pinkerton SD. Can increasing awareness of HIV seropositivity reduce, J Acquir Immune Defic Syndr, 2007, vol. 44 (pg. 360-363). 10.1097/QAI.0b013e31802ea4dd PMC2410041 17159653

[14] Ji Y, Liang P, Shen J (2018). Risk factors affecting the mortality of HIV-infected patients with pulmonary tuberculosis in the cART era: a retrospective cohort study in China. Infect Dis Poverty.

[15] Jiang J, Qin X, Liu H (2019). An optimal BMI range associated with a lower risk of mortality among HIV-infected adults initiating antiretroviral therapy in Guangxi, China. Sci Rep.

[16] Johannessen A, Naman E, Ngowi BJ (2008). Predictors of mortality in HIV-infected patients starting antiretroviral therapy in a rural hospital in Tanzania. BMC Infect Dis.

[17] Juffermans NP, Speelman P, Verbon A (2001). Patients with active tuberculosis have increased expression of HIV coreceptors CXCR4 and CCR5 on CD4(+) T cells. Clin Infect Dis.

[18] Kalsdorf B, Skolimowska KH, Scriba TJ (2013). Relationship between chemokine receptor expression, chemokine levels and HIV-1 replication in the lungs of persons exposed to Mycobacterium tuberculosis. Eur J Immunol.

[19] Kirby DB, Laris BA, Rolleri LA (2007). Sex and HIV education programs: their impact on sexual behaviors of young people throughout the world. J Adolesc Health..

[20] Liu E, Spiegelman D, Semu H, Hawkins C, Chalamilla G, Aveika A, Nyamsangia S, Mehta S, Mtasiwa D, Fawzi W (2011). Nutritional Status and Mortality Among HIV-Infected Patients Receiving Antiretroviral Therapy in Tanzania. J Infect Dis.

[21] Maokola W, Ngowi B, Lawson L, Mahande M, Todd J, Msuya SE (2020). Performance of and factors associated with tuberculosis screening and diagnosis among people living with HIV: Analysis of 2012-2016 Routine HIV Data in Tanzania. Front Public Health.

[22] Mollel E, Lekule I, Lynen L, Decroo T (2019). Effect of reliance on Xpert MTB/RIF on time to treatment and multidrug-resistant tuberculosis treatment outcomes in Tanzania: a retrospective cohort study. Int Health.

[23] Mollel EW, Chilongola JO, Mpagama SG, Kibiki GS (2017). Evaluation of XpertMTB/Rif performance for diagnosis of tuberculosis among HIV positive patients in northern Tanzania. Tanzan J Health Res..

[24] Mollel EW, Maokola W, Todd J, Msuya SE, Mahande MJ (2019). Incidence rates for tuberculosis among HIV infected patients in Northern Tanzania. Front Public Health.

[25] NACP (2017). National Guidelines for the Management of HIV and AIDS, Sixth Edition.

[26] Nakata K, Rom WN, Honda Y (1997). Mycobacterium tuberculosis enhances human immunodeficiency virus-1 replication in the lung. Am J Respir Crit Care Med.

[27] Narayanan S, Swaminathan S, Supply P (2010). Impact of HIV infection on the recurrence of tuberculosis in South India. J Infect Dis.

[28] Sabasaba A, Mwambi H, Somi G, Ramadhani A, Mahande MJ (2019). Effect of isoniazid preventive therapy on tuberculosis incidence and associated factors among HIV infected adults in Tanzania: a retrospective cohort study. BMC Infect Dis..

[29] Setegn T, et al. Predictors of mortality among adult antiretroviral therapy users in Southeastern Ethiopia :retrospective cohort study. AiIDS Res Treatment, Hindawi Publishing Corporation. 2015;148769. 10.1155/2015/148769. 10.1155/2015/148769 PMC4364127 25821596

[30] Seyoum D, et al. Risk factors for mortality among adult HIV/AIDS patients following antiretroviral therapy in Southwestern Ethiopia : an assessment through survival models. Int J Env Res Public Health. 2017:1–12. 10.3390/ijerph14030296. 10.3390/ijerph14030296 PMC5369132 28287498

[31] Sieleunou, I,Souleymanou M, Schonenberger AM, Menten J, Boelaert M. Determinants of survival in AIDS patients on antiretroviral therapy in a rural centre in the Far North Province, Cameroon, Trop Med Int Health, 2009, 14 (pg. 36-43). 10.1111/j.1365-3156.2008.02183.x 19017309

[32] Sileshi B (2013). Predictors of mortality among TB-HIV co-infected patients being treated for tuberculosis in Northwest Ethiopia : a retrospective cohort study. BMC Infect Dis.

[33] Sonnenberg P, Murray J, Glynn JR, Shearer S, Kambashi B, Godfrey-Faussett P (2001). HIV-1 and recurrence, relapse, and reinfection of tuberculosis after cure: a cohort study in South African mineworkers. Lancet.

[34] Steingart KR, Schiller I, Horne DJ (2014). Xpert ® MTB/RIF assay for pulmonary tuberculosis and rifampicin resistance in adults. Cochrane Database Syst Rev..

[35] Teklu AM, et al. Factors associated with mortality of TB/HIV co-infected patients in Ethiopia factors associated with mortality of TB/HIV co-infected patients in Ethiopia. Ethiop J Health Sci. 2017;27(1). 10.4314/ejhs.v27i1.4S. 10.4314/ejhs.v27i1.4s PMC5402803 28465651

[36] Toossi Z, Johnson JL, Kanost RA (2001). Increased replication of HIV-1 at sites of Mycobacterium tuberculosis infection: potential mechanisms of viral activation. J Acquir Immune Defic Syndr.

[37] UNAIDS (2016). Global AIDS Updates 2016.

[38] UNAIDS (2019). UNAIDS DATA 2019.

[39] WHO. WHO policy on collaborative TB/HIV activities: guidelines for national programmes and other stakeholders. In: WHO Policy on Collaborative TB/HIV Activities: Guidelines for National Programmes and Other Stakeholders: WHO; 2012. 23586124

[40] WHO (2013). Policy update: Xpert MTB/RIF assay for the diagnosis of pulmonary and extrapulmonary TB in adults and children.

[41] WHO. Global Tuberculosis Report 2018: World Health Organization; 2018.

[42] WHO. Global Tuberculosis Report 2019: World Health Organization (2019); 2019.

